# Increase of electronegative-LDL-fraction ratio and IDL-cholesterol in chronic kidney disease patients with hemodialysis treatment

**DOI:** 10.1186/1476-511X-11-111

**Published:** 2012-09-10

**Authors:** Yuji Hirowatari, Yasuhiko Homma, Joe Yoshizawa, Koichiro Homma

**Affiliations:** 1 Bioscience Division, TOSOH CORPORATION, 2743-1 Hayakawa Ayase-shi, Kanagawa, 252-1123, Japan; 2 Hiratsuka Lifestyle-related Disease and Homedialysis Clinic, Hiratsuka, Japan; 3 Department of Clinical Health Science, Tokai University School of Medicine, Isehara, Japan; 4 Department of International Medicine, School of Medicine, Keio University, Shinjuku, Japan; 5 Department of Emargency Medicine, School of Medicine, Keio University, 35 Shinanomachi, Shinjuku, 160-8582, Japan

**Keywords:** Electronegative-LDL, IDL, Hemodialysis, Anion-exchange chromatographic method

## Abstract

**Background:**

It is known that the increased level of IDL and oxidized LDL are associated with risk of cardiovascular disease, and the lipoprotein abnormalities accelerate atherosclerosis. Cardiovascular disease is a major cause of mortality in chronic kidney disease patients with hemodialysis treatment (HD-Ps). Therefore, the estimation of lipoprotein profiles is important for prevention of cardiovascular disease in HD-Ps. We previously established an anion-exchange chromatographic method for measurement of cholesterol level in subclasses of HDL and LDL, IDL, VLDL, and chylomicron. An electronegative-LDL-fraction contained minimally oxidized-LDL. Lipoprotein profile can be accurately and conveniently determined by the new method.

**Finding:**

In this study, lipoprotein profiles in HD-Ps and age-matched healthy subjects were estimated by using our established anion-exchange chromatographic method. The ratio of electronegative-LDL-cholesterol to total LDL-cholesterol and IDL-cholesterol in HD-Ps were significant higher than those in healthy subjects.

**Conclusions:**

The results suggest that the ratio of electronegative-LDL-cholesterol to total LDL-cholesterol and IDL-cholesterol obtained by the new method may serve as useful markers for risk of cardiovascular disease in HD-Ps.

## Introduction

Cardiovascular disease is a major cause of mortality in chronic kidney disease patients with hemodialysis (HD-Ps) [1]. It is known that HDL and LDL are decreased, and IDL and VLDL were increased in end-stage renal disease [2]. The patients with chronic renal failure frequently have abnormality of lipoprotein metabolism, and the oxidized modification of LDL and HDL is one of the major metabolism abnormalities [3]. We previously reported an anion-exchange chromatographic method (AEX-HPLC) for measurement of cholesterol level in two HDL-subclasses, two LDL-subclasses, IDL, VLDL, and chylomicron, and the cholesterol levels of electronegative-LDL-fraction, which is eluted later from the column, in patients with coronary artery disease were increased [4]. The minimally oxidized-LDL (MM-LDL) prepared by incubating with 2 μmol/L copper ion for 4 hours at 37 °C *in vitro*[5] was eluted at the position of electronegative-LDL [4].

In this study, lipoprotein profiles in HD-Ps and age-matched healthy subjects were estimated by using our established AEX-HPLC, and those in HD-Ps were compared to those in healthy subjects.

## Methods

The study samples were obtained from HD-Ps (n = 25) and the age-matched healthy controls (n = 25) after 12 hour-overnight fast. The HD-Ps were undergoing 3 times a week, 4 hours hemodialysis treatment using high-flux polysulphone dialysis membranes and standard heparin dose for anti-coagulation. This study was approved by the ethics committee at the Tokai University, and written informed consent was obtained from all participants.

The used AEX-HPLC was previously reported [4]. The major components of earlier- and later-eluting fraction of HDL (nonelectronegative-HDL and electronegative-HDL) were HDL3 and HDL2, respectively. The earlier-eluting subfraction of LDL (nonelectronegative-LDL) was changed into the later-eluting subfraction of LDL (electronegative-LDL) after oxidized by incubating with copper ion *in vitro*. The values of coefficients of variation of each lipoprotein cholesterol level were as follows: within-day assay (nonelectronegative-HDL, 1.45-2.18 %; electronegative-HDL, 0.92-1.80 %; nonelectronegative-LDL, 0.85-2.54 %; electronegative-LDL, 2.08-7.64 %; IDL, 3.83-9.80 %; VLDL, 1.41-11.68 %; chylomicron, 8.44-19.23 %) and between-day assay (nonelectronegative-HDL, 3.60-3.98 %; electronegative-HDL, 2.69-4.85 %; nonelectronegative-LDL, 2.55-6.67 %; electronegative-LDL, 5.08-7.62 %; IDL, 6.58-12.10 %; VLDL, 5.69-21.88 %; chylomicron, 20.19-23.50 %) [4].

GFR was estimated using the equation proposed by the Japanese society of nephrology in 2009: estimated GFR (mL/min/1.73 m^2^) = 194 x (creatinine)^-1.094^ x (age)^-0.287^ (x 0.739 if female).

The data are presented as the mean + standard deviation (SD). Student’s t-test or Mann-Whitney U-test was used to compare variables between HD-patients and healthy controls. P values <0.05 were considered significant.

## Results

Table 1 shows the data of characteristics and lipoprotein profiles in HD-Ps and healthy subjects. Age, sex and triglyceride between HD-Ps and healthy subjects were not significantly different. Total cholesterol, LDL-cholesterol, HDL-cholesterol and estimated GFR in HD-Ps were significantly lower than those in healthy subjects. The cholesterol levels in nonelectronegative-HDL and nonelectronegative-LDL were lower, and IDL-cholesterol and the ratio of electronegative-LDL-cholesterol to total LDL-cholesterol (nonelectronegative-LDL + electronegative-LDL) were higher in HD-Ps than in healthy controls.

**Table 1:** Charactaristics and lipoprotein profiles of hemodialysis patients and healthy controls

	**Hemodialysis patients**	**Healthy controls**	***P *value**
	(n = 25)	(n = 25)	
Age (years)	63 ± 13	63 ± 3	NS
Sex (male/female)	10/15	15/10	NS
Duration of hemodialysis (years)	1.9 ± 1.0		
BMI (kg/m2)	21 ± 3	22 ± 2	NS
Total cholesterol (mmol/L)	4.28 ± 1.02	5.07 ± 0.30	<0.005
Triglyceride (mmol/L)	1.00 ± 0.35	0.86 ± 0.31	NS
LDL cholesterol (mmol/L)	2.22 ± 0.72	3.00 ± 0.33	<0.0001
HDL cholesterol (mmol/L)	1.29 ± 0.43	1.68 ± 0.32	<0.001
Creatinine(mmol/L)	0.810 ± 0.204	0.061 ± 0.010	<0.0001
Estimated GFR (mL/min/1.73 m^2^)	4.1 ± 1.9	69.6 ± 15.3	<0.0001
Anion-exchange chromatgraphic method			
Nonelectronegative-HDL cholesterol (mmol/L)	0.54 ± 0.13	0.82 ± 0.11	<0.0001
Electronegative-HDL cholesterol (mmol/L)	0.86 ± 0.36	0.75 ± 0.26	NS
Nonelectronegative-LDL cholesterol (mmol/L)	2.29 ± 0.78	2.87 ± 0.30	<0.05
Electronegative-LDL cholesterol (mmol/L)	0.32 ± 0.13	0.34 ± 0.09	NS
IDL cholesterol (mmol/L)	0.099 ± 0.040	0.074 ± 0.035	<0.05
VLDL cholesterol (mmol/L)	0.23 ± 0.12	0.20 ± 0.10	NS
Chylomicron cholesterol (mmol/L)	0.075 ± 0.037	0.077 ± 0.063	NS
Electronegative-LDL-s / total LDL (%)	12.5 ± 3.4	10.4 ± 2.2	<0.05

NS indicates not significant.

## Discussion

Shoji et al. reported that the cholesterol levels of HDL and LDL were lower, and IDL and VLDL were higher in end-stage renal disease [2]. Furthermore, the IDL-cholesterol level was the best lipoprotein parameter that was most closely associated with aortic stiffness in HD-Ps [2]. We showed the lower cholesterol levels of HDL and LDL, and the higher IDL-cholesterol level in the patients with end-stage renal disease on hemodialysis, and that the VLDL-cholesterol in the patients was increased, but not significantly (Table 1). Shoji et al. also propose that the IDL-cholesterol level is a target in the management of dyslipidemia on renal disease, but the problem is that it is difficult to measure IDL-cholesterol levels by ultracentrifugation in routine clinical practice [2]. Our established AEX-HPLC is a convenient method to measure IDL-cholesterol levels. In the method, a small sample volume of serum (4.5 μL) is injected into a column, and lipoprotein classes in serum sample are separated and eluted by a step-gradient of ion concentrations in order. The post-column elute is reacted with an enzymatic reagent which contains cholesterol esterase and cholesterol oxidase. The assay time of one sample is merely 23 minutes. We thought that the AEX-HPLC for measurement of cholesterol levels of LDL- and HDL-subclasses, IDL, VLDL, and chylomicron may be informative for the management of dyslipidemia in HD-Ps.

Miida et al. reported the higher level of preβ1-HDL and the lower level of HDL3 which is a subfraction of α-migrating HDL in HD-Ps, and that the conversion of lecithin: cholesterol acyltransferase of preβ1-HDL to α-migrating HDL is delayed in HD-Ps [6]. We showed that nonelectronegative-HDL level of which major component is HDL3 was decreased in HD-Ps (Table 1). Therefore, the cholesterol level of nonelectronegative-HDL may be informative for the management of dyslipidemia in HD-Ps.

Oxidized LDL is generally considered to play an important role in the development of atherosclerosis. It was previously reported that the production of oxidized LDL and oxidized HDL were increased in chronic renal failure [3]. Additionally, it was reported that oxidized LDL/total LDL ratio is higher in HD-Ps with vascular calcification [7]. We indicated that electronegative-LDL-cholesterol/total LDL-cholesterol ratio (nonelectronegative-LDL + electronegative-LDL) ratio in HD-Ps was increased (Table 1).

In conclusion, the present study indicates that electronegative-LDL/total LDL (nonelectronegative-LDL + electronegative-LDL) ratio and IDL-cholesterol obtained by the AEX-HPLC may serve as useful makers for risk of cardiovascular disease in HD-Ps.

## Findings

In this study, the lipoprotein profiles were estimated with chronic kidney disease patients with hemodialysis treatment (HD-Ps) by using our developed anion-exchange chromatographic method. The ratio of electronegative-LDL-cholesterol to total LDL-cholesterol and IDL-cholesterol in HD-Ps were significant higher than those in healthy subjects. The results suggested that the ratio of electronegative-LDL-cholesterol to total LDL-cholesterol and IDL-cholesterol obtained by the new method may serve as useful markers for risk of cardiovascular disease in HD-Ps.

## Abbreviations

HD-Ps: chronic kidney disease patients with hemodialysis treatment; AEX-HPLC: anion-exchange chromatographic method.

## Competing interests

There were no conflicts of interest in this study.

## Authors’ contributions

YH contributed to the study conception and design, and carried out data acquisition, data analysis and interpretation. YH also wrote paper. YHo contributed to the study conception and design, and carried out data acquisition, data analysis and interpretation. JY carried out data acquisition. KH contributed to the study conception and design. All authors read and approved the final manuscript.
